# Accelerated Compressed Sensing Based CT Image Reconstruction

**DOI:** 10.1155/2015/161797

**Published:** 2015-06-18

**Authors:** SayedMasoud Hashemi, Soosan Beheshti, Patrick R. Gill, Narinder S. Paul, Richard S. C. Cobbold

**Affiliations:** ^1^Institute of Biomaterials and Biomedical Engineering, University of Toronto, Toronto, ON, Canada M5S 3G9; ^2^Department of Electrical and Computer Engineering, Ryerson University, Toronto, ON, Canada M5B 2K3; ^3^Rambus Inc., Sunnyvale, CA 94089, USA; ^4^Joint Department of Medical Imaging, Toronto General Hospital, University Health Network, Toronto, ON, Canada M5G 2C4

## Abstract

In X-ray computed tomography (CT) an important objective is to reduce the radiation dose without significantly degrading the image quality. Compressed sensing (CS) enables the radiation dose to be reduced by producing diagnostic images from a limited number of projections. However, conventional CS-based algorithms are computationally intensive and time-consuming. We propose a new algorithm that accelerates the CS-based reconstruction by using a fast pseudopolar Fourier based Radon transform and rebinning the diverging fan beams to parallel beams. The reconstruction process is analyzed using a maximum-a-posterior approach, which is transformed into a weighted CS problem. The weights involved in the proposed model are calculated based on the statistical characteristics of the reconstruction process, which is formulated in terms of the measurement noise and rebinning interpolation error. Therefore, the proposed method not only accelerates the reconstruction, but also removes the rebinning and interpolation errors. Simulation results are shown for phantoms and a patient. For example, a 512 × 512 Shepp-Logan phantom when reconstructed from 128 rebinned projections using a conventional CS method had 10% error, whereas with the proposed method the reconstruction error was less than 1%. Moreover, computation times of less than 30 sec were obtained using a standard desktop computer without numerical optimization.

## 1. Introduction

Compared to conventional radiography, CT results in a relatively large radiation dose to patients, which is of serious long-term concern in its potential for increasing the risk of developing cancer [1, 2]. As a result, low dose CT imaging that maintains the resolution and achieves good contrast to noise ratio has been the goal of many CT developments over the past decade. However, low dose CT images reconstructed with conventional filtered back projection (FBP), which directly calculates the image in a single reconstruction step, suffer from low contrast to noise ratios. Iterative reconstruction approaches, namely, Algebraic Reconstruction Technique (ART) [3–5] and statistical iterative reconstruction (SIR) [6, 7], have been proposed to improve the reconstruction quality and to decrease image artifacts. The iterative algorithms improve the quality by considering more accurate models for the CT images and geometries. However, they significantly increase the computational complexity, compared to the FBP based methods.

Iterative reconstruction methods have progressed with the introduction of compressed sensing (CS) [8, 9]. Such methods are capable of reconstructing high quality images from a substantially smaller number of views than those needed in FBP [10], thereby permitting the use of a much lower dose scanning protocol than that needed in conventional reconstruction methods. However, conventional CS-based CT reconstructions are computationally expensive and the statistics of CT measurements are not usually incorporated in the problem formulation [11–16].

In this paper, we propose a fast weighted CS-based CT reconstruction algorithm, the weights of which are direct consequences of the geometry and the CT statistics. The first part of this paper leads to the proposed weighted CS formulation, which is solved by a computationally efficient method discussed in the second part.

## 2. CS-Based CT Reconstruction and Its Challenges

Compressed sensing prescribes solving the *ℓ*_1_ optimization problem that can be represented by

(1) $$\begin{array}{c} \hat{x}=\underset{x}{\arg\min}\frac{1}{2}{\left\|\;y-Ax\;\right\|}_{2}^{2}+\mu {\left\|\;W^{T}x\;\right\|}_{1} \end{array}$$

or other similar forms, for example, the *ℓ*_1_ norm of the image gradient, such as

(2) $$\begin{array}{c} \hat{x}=\underset{x}{\arg\min}\frac{1}{2}{\left\|\;y-Ax\;\right\|}_{2}^{2}+\mu TV\left(\;f\;\right), \end{array}$$

to recover a sparse signal from few samples. In these equations, *μ* acts as a regularization parameter specifying a trade-off between the image prior model and the fidelity to observations, **A** is the measurement matrix, **x** is the column vector representation of the desired image (*f*), **y** is the measured data, *W* ^*T*^ is a sparsifying transform, ‖*x*‖_*q*_ = (∑_*i*_|*x*_*i*_|^*q*^)^1/*q*^, and **T** **V** denotes the total variation $TV(f)=\sum_{i}\sqrt{{\left(\nabla_{x}f\right)}_{i}^{2}+{\left(\nabla_{y}f\right)}_{i}^{2}}$, where ∇_*x*_ and ∇_*y*_ are the first derivatives in the *x* and *y* directions of the desired image.

The main challenge in solving these optimization problems within a reasonable amount of time arises from the size of the measurement matrix **A**. Currently, in most available CS-based reconstruction methods, the measurement matrix **A** is a projection matrix which models the rays going through the patient. To reconstruct a 512 × 512 pixel image from 900 sensors and 1200 projection angles, **A** would be a 1080000 × 262144 matrix. Although this matrix is sparse, each iteration typically requires two multiplications by **A** and **A** ^**T**^, resulting in a very significant increase in the computation burden for reconstructing a 512 × 512 image [11, 12] as compared to FBP based methods. To enable the CS-based CT reconstruction to be done in a reasonable computation time, GPU based algorithms have been proposed [17].

### 2.1. Complexity Reduction Using the Pseudopolar Fourier Transform (PPFT)

To reduce the computational burden on the Radon transform, the central slice theorem (CST) or direct Fourier reconstruction (DFR) has been used [18]. This relates the 1D Fourier transform of the projections to the 2D Fourier transform of the image. Such a method requires the interpolation of polar data onto a Cartesian grid followed by an inverse FFT on the same grid to reconstruct the CT image. Since interpolation does not have a known analytical adjoint, its use in iterative algorithms is not a practical option. In addition, inclusion of a gridding and regridding step at each iteration increases the overhead computational complexity. This problem has been extensively studied in non-Cartesian magnetic resonance imaging reconstruction algorithms [19].

**Figure 1:**
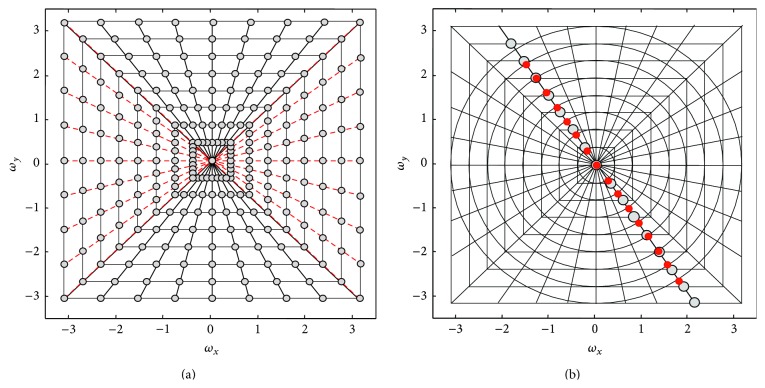
(a) Pseudopolar grids: red lines are basically horizontal (BH) and the black lines are basically vertical (BV). (b) Polar grids (red dots) on the pseudopolar grids (gray dots).

An equally sloped tomography (EST) method was originally proposed for electron beam tomography [20–22] to improve the DFR-based algorithms. EST is an iterative method that makes use of the pseudopolar Fourier transform (PPFT) [23]. It calculates the Fourier coefficients of an image directly on pseudopolar grids, which contain two types of samples: basically horizontal (BH) and basically vertical (BV), as can be seen in Figure 1. To reconstruct an *N* × *N* image from its PPFT coefficients, 4*N* ^2^ samples are needed (2*N* samples on 2*N* equally sloped radial lines). A fast algorithm has been proposed by Averbuch et al. [23] to calculate the PPFT and its adjoint with complexity of *O*(*N* ^2^log*N*). This algorithm can then be used to implement a fast and efficient 2D Radon transform on the equally sloped radial lines. The PPFT has three important properties which makes it a good alternative to conventional DFR methods: (1) it is closer to a polar (equiangular line) grid than to a Cartesian grid, which significantly decreases the gridding error, (2) it has both a fast forward and a fast backward calculation algorithm [23], which enables our proposed algorithm to avoid the regridding step used in iterative non-Cartesian Fourier based reconstruction methods, and (3) it has an analytical adjoint function. As a result, it can efficiently be used in iterative algorithms, including compressed sensing [24, 25]. However, it should be noted that Fourier-based reconstruction algorithms, for example, ESR and DFR, are only valid for parallel X-ray projections.

A major objective of this paper is to accelerate the CS-based CT reconstruction by decreasing the CS complexity using PPFT-based Radon transform proposed in [26]. The application of the proposed method is extended to equiangular parallel and nonparallel geometries using rebinning.

#### 2.1.1. Rebinning Process

To enable use of the PPFT-based Radon transform for nonparallel geometries, the projected rays must first be transformed to parallel beams [27]. This requires two interpolation steps. At first, projections are interpolated on equally sloped radial lines on the following angles:

(3) φBH=tan−12mN,−N2≤m<N2,φBV=tan−12mN+π2,−N2≤m<N2.

**Figure 2:**
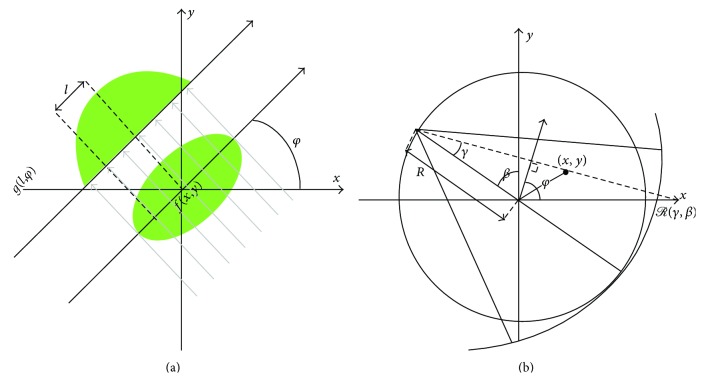
(a) Parallel beam geometry and (b) fan beam geometry with a curved detector.

This step makes use of the following relationships between fan and parallel beams:

(4) Rγ,β=gRsin⁡γ,β+γ,l=Rsin⁡γ,φ=β+γ,

where *γ*, *R*, *φ* = *φ*_BH_ ∪ *φ*_BV_, and *β* are geometry parameters defined in Figure 2. Moreover, *ℛ*(*γ*, *β*) is the fan beam projected data and *g*(*R*sin⁡*γ*, *β* + *γ*) is the corresponding rebinned parallel ray. In the second interpolation step, the radial samples are interpolated on the pseudopolar grids shown in Figure 1(b). To reduce the interpolation error, these radial lines are zero-padded and the 1D Fourier transforms of the zero-padded radial lines are interpolated. This is equivalent to oversampling in the Fourier domain, which makes the interpolation error manageably small.

### 2.2. Measurement Noise and CS-Based CT Reconstruction

The measurement noise in CT scanners can best be modeled by a Poisson distribution [27], while the noise that is considered in classical CS formulations, such as those given by (1) and (2), is white additive Gaussian noise [8, 9]. Therefore, to enable a more accurate low dose CS-based CT reconstruction, the classical CS formulations should be modified. To address this problem in prior studies different approaches have been used. For instance, in [14, 28, 29] to account for the statistical properties associated with low-dose measurements, an iterative SIR based technique followed by TV denoising was used. The penalized weighted least squares (PWLS) formulation of statistical CT reconstruction was used in [30, 31] to improve the quality of reconstructed images. This PWLS formulation can be characterized as a weighted *ℓ*_1_ minimization problem as proposed by Candès et al. [32], who showed that by using appropriate weights the quality of the recovered signal can be improved. In this modified CS formulation, the weights could account for the statistical characteristics of the signals.

In this paper, a fast CS-based CT reconstruction is proposed using a pseudopolar based Radon transform to decrease the complexity of the CS recovery. The method requires the projections to be rebinned on equally sloped lines. We propose a weighted CS formulation in the framework of statistical CT image reconstruction algorithms. The weight, denoted by error adaptation weight (EAW), is a function of the rebinning interpolation error [25] and the Poisson noise of the CT projections [31] calculated from a maximum a posteriori (MAP) model of CT reconstruction, described in next section.

## 3. Maximum a Posteriori (MAP) Model of CT

X-ray projections of the parallel beam CT can be expressed as the Radon transform of the object. The Radon transform is defined as [33]

(5) gl,φ=∫−∞∞∫−∞∞fx,yδxcos⁡φ+ysin⁡φ−ldx dy,

which is the integral along a ray at angle *φ* and at the distance *l* from the origin, *δ*(*x*, *y*) is Dirac delta function, and *f*(*x*, *y*) is the object attenuation at (*x*, *y*). However, this is not what the scanners directly measure. Scanner detectors measure the number of photons that hit the detector, *λ*(*l*, *φ*), which is usually modeled by Poisson distribution with expected value of $\bar{\lambda }(l,\varphi )$ [6, 27]. The relation between *g*(*l*, *φ*) and $\bar{\lambda }(l,\varphi )$ is $g(l,\varphi )=-log\left(\bar{\lambda }(l,\varphi )/\lambda_{T}\right)$, where *λ*_*T*_ is the number of radiated photons from the X-ray source. It should be noted that *λ*(*l*, *φ*) is usually corrupted with two kinds of noise: electrical noise of the detectors (with variance of *σ*_*n*_^2^) and the photon counting noise (observed counts are drawn from a Poisson distribution of mean $\bar{\lambda }$). If we consider the discrete formulation in which **y** denotes the vectorized *g*(*l*, *φ*), **x** denotes the vectorized *f*(*x*, *y*), and **A** is the projection matrix, using the second order Taylor series expansion of the Poisson distribution, the log likelihood of the measurements is given by [34, 35]

(6) $$\begin{array}{c} \log p\left(\;y\;|\;x\;\right)\approx -\frac{1}{2}{\left(\;y-Ax\;\right)}^{T}D\left(\;y-Ax\;\right)+O\left(\;y^{3}\;\right), \end{array}$$

in which *O*(**y** ^3^) is a function which depends upon measured data only and *D* is a diagonal matrix. For the purpose of the MAP estimation *O*(**y** ^3^) may be ignored since it does not depend on **x**. Ignoring this term, (6) describes a simplified CT model that can be written as

(7) $$\begin{array}{c} y=Ax+n, \end{array}$$

in which **n** is Gaussian distributed noise with a covariance matrix *D* ^−1^ and *d*_*i*_, the *i*th diagonal element of *D*, is proportional to the detector counts, corresponding to the maximum likelihood of the inverse of the variance of the projection measurements, that is, to 1/*σ*_*y*_*i*__^2^. The *i*th measured projection **y**_*i*_ is given by

(8) yilog⁡λTλi=log⁡λTλ¯i+log⁡λ¯iλi≈y¯i+1−λiλ¯i,

where ${\bar{y}}_{i}$ is noiseless and *λ*_*i*_ follows the Poisson distribution with $\sigma_{\lambda_{i}}^{2}={\bar{\lambda }}_{i}$. As a result the variance of projection data can be estimated from $\sigma_{y_{i}}^{2}\approx (\sigma_{\lambda_{i}}^{2}+\sigma_{n}^{2}){\left({\bar{\lambda }}_{i}\right)}^{-2}$. Using *λ*_*i*_ as an unbiased estimation of ${\bar{\lambda }}_{i}$, the diagonal elements of *D* can be expressed as

(9) $$\begin{array}{c} d_{i}=\frac{1}{\sigma_{y_{i}}^{2}}=\frac{\lambda_{i}^{2}}{\sigma_{n}^{2}+\lambda_{i}}. \end{array}$$

To reconstruct the image from the projections, the MAP estimator can be used:

(10) $$\begin{array}{c} \hat{x}=\underset{x}{\arg\max}\log p\left(\;y\;|\;x\;\right)+h\left(\;x\;\right). \end{array}$$

Here *h*(*x*) = log⁡*p*(**x**) acts as a penalty function, which is used to statistically model the wavelet coefficients distribution and the piecewise constant (locally constant) nature of CT images.

### 3.1. Piecewise Constant and Sparsity of the CT Images in MAP Model

Many studies [36, 37] have shown that the wavelet transform of a variety of images, *θ* = *W* ^*T*^ **x**, can be modeled by generalized Gaussian distribution (GGD), that is, by

(11) $$\begin{array}{c} p\left(\;\theta_{i}\;\right)=K\left(\;s,q\;\right)\cdot \exp\left(\;-{\left|\;\frac{\theta_{i}}{s}\;\right|}^{q}\;\right), \end{array}$$

where *θ* is the wavelet coefficients of the image **x** = *Wθ*, *W* is inverse wavelet transform, *s* and *q* are the parameters of the GGD, and *K*(*s*, *q*) is the normalization parameter. It should be noted that when *q* = 1, the GGD is equivalent to Laplacian distribution and when *q* = 2, it describes a Gaussian distribution. Using (6), (10), and (11), the MAP model for CT images can be expressed as

(12) $$\begin{array}{c} \hat{x}=\underset{x}{\arg\max}\frac{1}{2}{\left\|\;y-Ax\;\right\|}_{D}^{2}+\mu {\left\|\;W^{T}x\;\right\|}_{q}, \end{array}$$

where ‖**y** − **A** **x**‖_*D*_^2^ = (**y** − **A** **x**)^*T*^ *D*(**y** − **A** **x**), *μ* is a function of GGD parameters, and *q* is typically in the range 0 < *q* ≤ 1. Another prior on *p*(*x*) is that the objects being imaged are piecewise constant. A *ρ* variation distribution can be used to describe the piecewise constant functions [38]. If *x*_*n*_(*t*) = ∑_*j*=1_^*n*^ *x*_*j*_^*n*^Ψ_*j*_^*n*^(*t*) is a piecewise function spanned by the roof-top basis Ψ_*j*_^*n*^(*t*), the following class of probability distribution can be used to describe it:

(13) px1n,…,xnn=υρ,nexp⁡−ann+11−ρ∑j=1n+1xjn−xj−1nρ,

where *a*_*n*_ > 0, *x*_0_^*n*^ = *x*_*n*+1_^*n*^ = 0, *υ*_*ρ*,*n*_ is normalizing factor, and [*x*_1_^*n*^,…, *x*_*n*_^*n*^]^*T*^ is a *ℝ* ^*n*^-valued random vector. When *ρ* = 1, this yields the total variation norm. Using *ρ* = 1 in (13), (10) becomes the following MAP model for CT images:

(14) $$\begin{array}{c} \hat{x}=\underset{x}{\arg\min}\frac{1}{2}{\left\|\;y-Ax\;\right\|}_{D}^{2}+\mu TV\left(\;f\;\right). \end{array}$$

As can be seen, (14) and (12) are generalized forms of the CS models given by (2) and (1), respectively. It has been shown that the quality of the reconstructed image can be improved by combining the sparsity and total variation penalty terms [39]. Therefore, both penalty functions were used in the proposed method to reconstruct CT images from the undersampled data, that is, from

(15) $$\begin{array}{c} \hat{x}=\underset{x}{\arg\min}\frac{1}{2}{\left\|\;y-Ax\;\right\|}_{D}^{2}+\mu_{1}{\left\|\;W^{T}x\;\right\|}_{q}+\mu_{2}TV\left(\;f\;\right), \end{array}$$

where *μ*_1_ and *μ*_2_ are regularization parameters.

## 4. Proposed CS Formulation: Measurement Noise and Interpolation Error

In Section 3, it was shown that the MAP estimator of CT is a form of the weighted CS problem given by (15), in which the weight is a function of noise variance and is denoted by *D* in (9). To avoid the computational burden associated with the huge projection matrix of CS-based CT, our proposed algorithm makes use of the fast PPFT-based Radon transform described in Section 2.1. This not only accelerates the computations by reducing the computational complexity, but also substantially reduces the gridding error and eliminates the regridding step (since it has a fast backward calculation algorithm, there is no need to regrid the updated image to pseudopolar grids at each iteration).

As described in Section 2.1.1, the proposed method is generalized for use with nonparallel geometries by rebinning the X-ray beams onto equally sloped radial lines as given by (3). The rebinning step induces interpolation error to the measured data, which propagates in each iteration of CS-based CT reconstruction. This problem has not received much attention in the literature. Fahimian et al. [24] proposed an EST method for reconstructing fan beam and helical cone beam images, in which they overcome the rebinning interpolation problem at each iteration by using a nonlocal total variation minimization smoothing step. In the method proposed by Hashemi et al. [25], an *ℓ*_2_-TV optimization scheme was used to reconstruct the CT images from fan beam projections. To compensate for the interpolation error, a confidence matrix was added to the CS scheme, enabling control of the error propagation in successive iterations.

To control the interpolation error, we make use of a MAP model of the CT reconstruction process. Denoting the variance of interpolation error by *e*_*i*_, the variance of the measurements is

(16) $$\begin{array}{c} \sigma_{y_{i}}^{2}\approx \left(\;\sigma_{\lambda }^{2}+\sigma_{n}^{2}+e_{i}\;\right){\left(\;{\bar{\lambda }}_{i}\;\right)}^{-2}. \end{array}$$

Using (8), the effects of both the noise variance and interpolation error can be lumped together into the form of an error adaptation weight (EAW), denoted by a diagonal matrix **C** with diagonal elements:

(17) $$\begin{array}{c} c_{i}=\frac{\lambda_{i}^{2}}{\sigma_{n}^{2}+\lambda_{i}+e_{i}}. \end{array}$$

Assuming a nearest neighbor interpolation, the interpolation error is linearly dependent on the distance between the desired and the measured grids; that is, *e*_*i*_ = *ϵ*_*i*_ × *λ*_*i*_ in which *ϵ*_*i*_ ∈ [0, *∞*) models the interpolation distance. Consequently, if the dose at each projection is high enough to ignore the electric noise *σ*_*n*_, the EAW can be rewritten as

(18) $$\begin{array}{c} c_{i}=\frac{1}{\sigma_{y_{i}}^{2}+\epsilon_{i}\sigma_{y_{i}}^{2}}=\lambda_{i}\times \frac{1}{1+\epsilon_{i}}. \end{array}$$

Using this definition, our proposed CS formulation to reconstruct the CT images can be expressed by

(19) $$\begin{array}{c} \hat{x}=\underset{x}{\arg\min}\frac{1}{2}{\left\|\;y-Ax\;\right\|}_{C}^{2}+\mu_{1}{\left\|\;W^{T}x\;\right\|}_{q}+\mu_{2}TV\left(\;f\;\right). \end{array}$$

This implicitly models the effect of polar and nonparallel projections to pseudopolar gridding, which in turn affects the noise in the data. This error is considered to be linearly dependent on the interpolation distance, while it is typically smaller using more accurate interpolation methods, that is, in Kaiser-Bessel based interpolation [19]. Therefore, the proposed CS formulation can be thought of as a minimization of an upper bound of the pseudopolar rebinning error. In addition, **C** in this formulation acts similar to a Jacobi preconditioner and therefore could accelerate the convergence rate [40].

### 4.1. Calculation of *ϵ*

**Figure 3:**
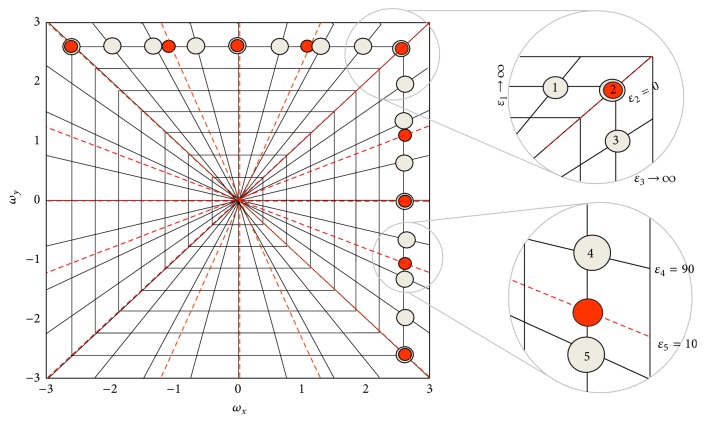
An example of calculating the interpolation error in the error adaptation weight (EAW).

The value of *ϵ*_*i*_ represents the error of the interpolated samples. If the interpolated sample is close to the original measurements, the value of *ϵ*_*i*_ is small and the confidence about the interpolated value is high. If the angular distance of the measured data from the interpolated line is more than the angular difference of the equally sloped lines, the interpolation error is considered to be high (*ϵ*_*i*_ → *∞*): this follows from the fact that the distance of points on the line from the true measured values is maximal and therefore the error is maximal. Using (18), this condition corresponds to *c*_*i*_ → 0. The closer the equally sloped lines are to the rays on which the measurements are made, the smaller the interpolation error will be, so that *ϵ*_*i*_s on that line get closer to zero. Finally, if the desired equally sloped rays are exactly on the polar lines, the interpolation error *e*_*i*_ is zero, which is equivalent to *ϵ*_*i*_ = 0. This process is illustrated in Figure 3.

In practice, (1/(1 + *ϵ*_*i*_)) can be estimated by rebinning an all-ones matrix with the same size as the measured data onto the equally sloped radial angles followed by an interpolation on the pseudopolar grid. Note that this has to be calculated only once before the reconstruction.

## 5. Solving the Proposed CS Formulation

To solve the proposed formulation, a fast composite splitting algorithm (FCSA) [41–43] is used to decompose (19) into two simpler subproblems given by

(20) x^1=arg⁡minx⁡f1x,f1x=12y−AxC2+μ1WTxq,x^2=arg⁡minx⁡f2x,f2x=12y−AxC2+μ2TVf,

in which **y** is the measured data interpolated/rebinned on the equally sloped lines, **A** is the PPFT-based Radon transform, and **A** ^*T*^ is its adjoint. By calculating ${\hat{x}}_{1}$ and ${\hat{x}}_{2}$, the FCSA method proposes that the solution to the problem can be obtained by a linear combination of the solutions of the two subproblems; that is,

(21) $$\begin{array}{c} \hat{x}=\Delta {\hat{x}}_{1}+\left(\;1-\Delta \;\right){\hat{x}}_{2}, \end{array}$$

in which Δ = *f*_2_/(*f*_1_ + *f*_2_) is a function of the values of the objective functions of the two subproblems. Each of these subproblems can be solved by a subgradient-projection based method [44]. The pseudocode of the proposed recovery is shown in Algorithm 1, in which prox{*g*(*x*), *z*} = arg min_*x*_ *g*(*x*) + (1/2)‖*x* − *z*‖_2_^2^. To find ${\hat{x}}_{1}$, the optimization problem in step (2) of this algorithm can be solved by a wavelet soft thresholding algorithm [36]. Moreover, to calculate ${\hat{x}}_{2}$ in step (3), the split Bregman TV based denoising algorithm as proposed in [45] was used. Finally, to estimate ${\hat{x}}_{k}$ in the *k*th iteration, (21) was used.

**Figure 4:**
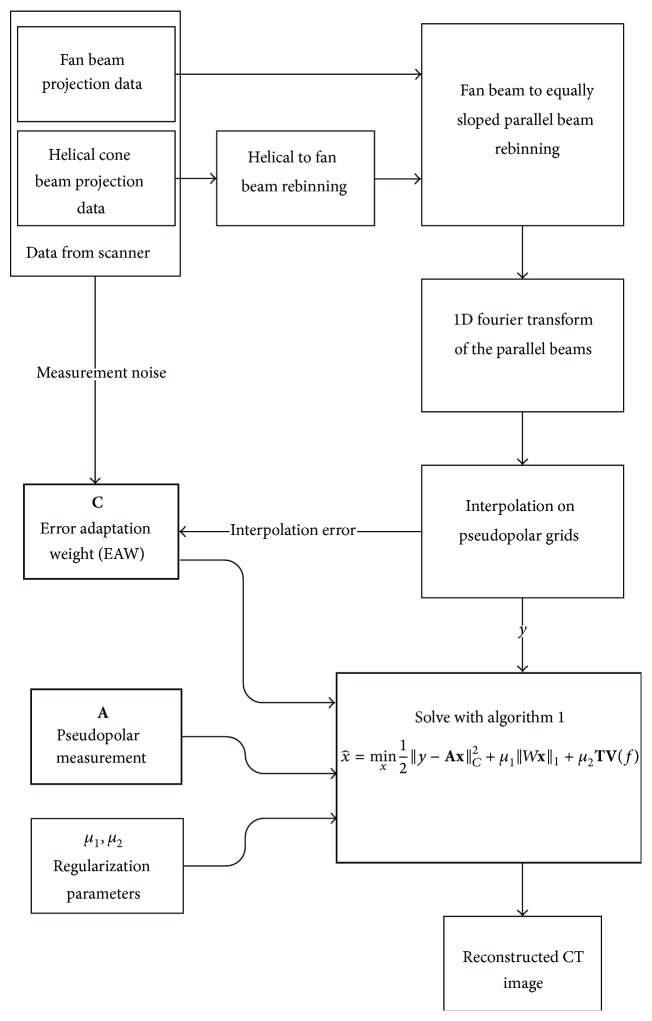
Flowchart of the proposed weighted CS-based CT reconstruction method.

In the proposed CT reconstruction algorithm, summarized in Figure 4, the Daubechies wavelets with four vanishing moments in 5 levels are used as the sparsifying transform *W*. The regularization parameters are manually tuned to *μ*_1_ = 0.05‖*W* ^*T*^(**A** ^*T*^ **y**)‖_*∞*_ and *μ*_2_ = 1 × 10^−3^ **T** **V**(**A** ^*T*^ **y**).

## 6. Simulation Methods

Fan beam simulations were performed using a Shepp-Logan phantom available in MATLAB (MathWorks, Massachusetts, USA), a custom made phantom that mimics different cardiac plaques and a clinical patient. This study was approved by our institutional (Toronto General Hospital, Toronto, ON, Canada) review board and individual patient consent was waived. X-ray projections of the phantoms and the patient were taken using a Toshiba Aquilion ONE^©^ scanner (Toronto General Hospital, Canada). The scanner gathers data from 900 projection angles in each 360° rotation. To be compatible with the available hardware, when the images were reconstructed from fewer than the 900 projections, the projection views were selected equiangularly. For all the scan protocols, the X-ray tube current-exposure time product was 50 mAs and the peak voltage was 120 kV. This current/voltage is high enough to ignore the electric noise in the simulations. Data from the central row of a volumetric scan on one single rotation served as the fan beam data.

## 7. Results and Discussion

In this section, the performance of the proposed method is evaluated in terms of the reconstruction time and accuracy. In addition, the effect of the EAW is evaluated in reducing the influence of the interpolation error and the Poisson measurement noise.

### 7.1. Reconstruction Time Acceleration

Compared to the other CS-based reconstruction techniques, the primary improvement of our proposed method is the major reduction in the computational burden. The reconstruction time of the proposed method is compared with a conventional ART-TV based CT reconstruction algorithm. The convergence of this method is justified by projection on convex set (POCS) algorithm. As an example in [46], an ART-TV based CT reconstruction method is proposed denoted by adaptive-steepest-decent POCS (ASD-POCS), which has been used by many other researchers [14, 29, 47, 48]. Here, we use a simple method in which the updates are calculated using an ART based method described below. This step is followed by a TV minimization step to project the updated image on a piecewise constant space. To solve the **y** = **A** **x** problem in ART step, a randomized Kaczmarz algorithm is used [4]. If **y** = **A** **x** is a linear system of equations and **x**_0_ is an arbitrary initial approximation to the solution, randomized Kaczmarz applies the following updating step at each iteration:

(22) $$\begin{array}{c} x_{k+1}=x_{k}+\frac{y_{r\left(i\right)}-\langle a_{r\left(i\right)},x_{k}\rangle }{{\left\|a_{r\left(i\right)}\right\|}_{2}^{2}}a_{r\left(i\right)}, \end{array}$$

where *r*(*i*) is chosen randomly from the set {1,2,…, *m*}, with probability proportional to ‖*a*_*r*(*i*)_‖_2_^2^, *a*_*i*_ is the *i*th row of **A**, and 〈·, ·〉 is the inner product of two vectors. Using this algorithm followed by the split Bregman TV minimization (denoted by SBROF in Algorithm 2), the ART-TV based method is described in Algorithm 2. In our simulations, the number of inner iterations used in Kaczmarz algorithm is 10 and the number of outer iterations is 50.

**Figure 5:**
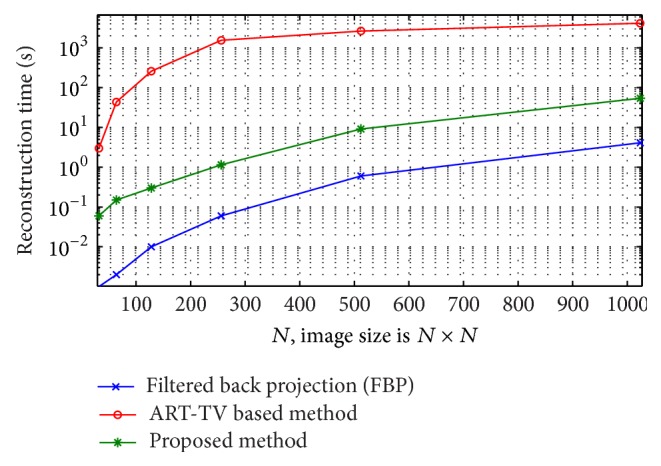
Reconstruction time comparison using a standard desktop computer, for (1) fan beam filtered back projection (FBP) reconstruction, (2) the proposed method, and (3) a fan beam ART-TV based method.

**Figure 6:**
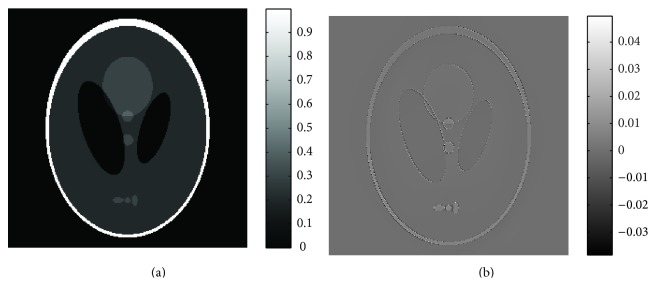
ART-TV reconstruction results: (a) Shepp-Logan phantom reconstructed from 200 projections and (b) reconstruction error.

Figure 5 compares the recovery time using (1) filtered back projection (FBP), (2) the proposed method, and (3) the ART-TV based method described in Algorithm 2(the ART method implementation is based on the codes provided in* algebraic iterative reconstruction methods (AIR Tools)* and* Tomobox* packages). It can be seen that for a 512 × 512 image the recovery time for the proposed method is approximatively 10–30 sec, using MATLAB on an Intel(R) Core(TM) i5 (3.2 GHz) CPU desktop PC with 16 GB of RAM.  Figure 6 shows the phantom reconstructed by ART-TV described in Algorithm 2 from 200 projections. The normalized reconstruction error ${\left\|x-\hat{x}\right\|}_{2}/{\left\|x\right\|}_{2}$ is 2 × 10^−2^.

### 7.2. Interpolation Error and Noise Correction Using EAW

**Figure 7:**
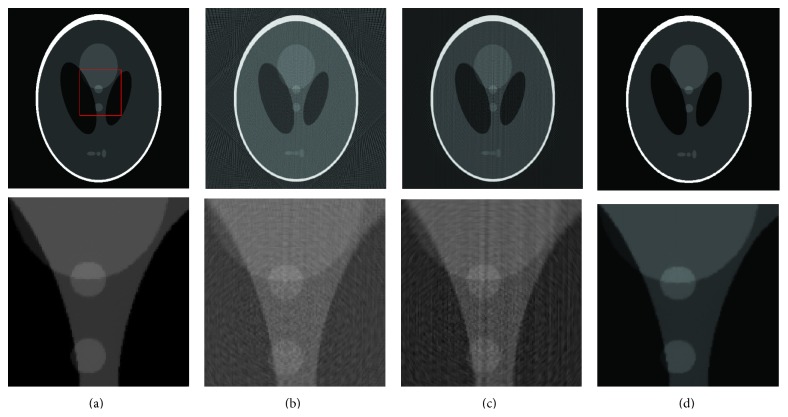
Simulation results for a Shepp-Logan phantom. The bottom row shows an expanded view of the region marked in panel (a). (a) Original phantom image. Reconstructions using 128 projections with (b) inverse PPFT-based Radon transform (normalized error ≈0.9), (c) an iterative soft thresholding based method without considering EAW (normalized error ≈10^−1^), and (d) the proposed method (normalized error ≈10^−2^). Rebinned parallel rays were used in all three methods to reconstruct the image.

**Figure 8:**
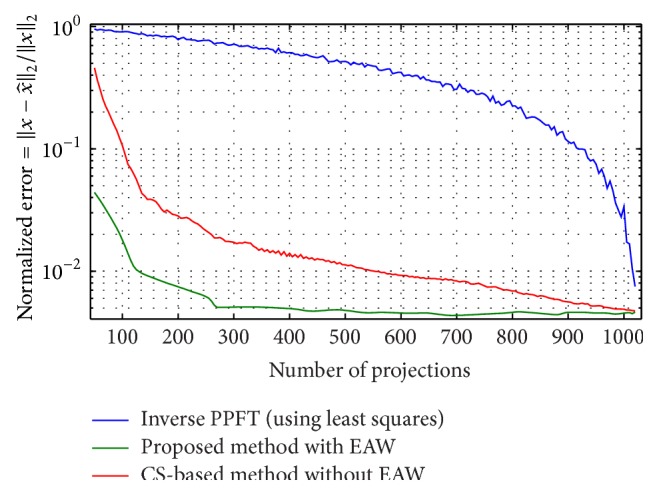
Normalized reconstruction error for the simulated Shepp-Logan phantom reconstructed with the inverse PPFT-based Radon transform, using an iterative soft thresholding based method without including the EAW and the proposed method. Rebinned parallel rays were used in all three methods to reconstruct the 512 × 512 image.

Equiangular fan beam projections of the Shepp-Logan phantom were computed on 128 projection views. This data was then rebinned to parallel rays on equally sloped angles as described by (3).  Figure 7 compares the reconstructed 512 × 512 images with the original image (1) using the inverse pseudopolar Fourier transform (least squares method), (2) without using EAW weights and they were solved using an iterative soft threshold-based method [49], and (3) using the proposed method with EAW. Note that the lower row consists of an expanded view of the marked central region, from which it is clear that the proposed method yields results very close to that shown in Figure 7(a). Based on the same phantom, Figure 8 compares the accuracy of the reconstruction error for all three methods as the number of projections is varied from 50 to 1024. Both of these figures show that the recovery accuracy is improved significantly by the inclusion of EAW to correct for rebinning errors. In particular, Figure 8 shows that the use of more than 300 projections for a 512 × 512 image does not significantly affect the reconstruction accuracy. Since the purpose of this particular study was to examine effects of EAW inclusion on rebinning interpolation error, noise was not included in the simulations.

**Figure 9:**
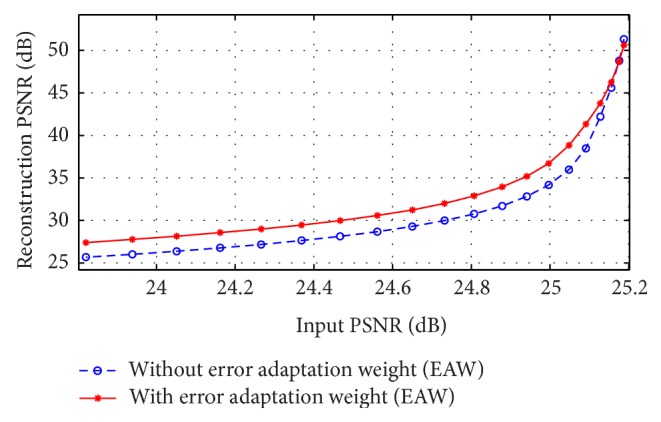
Effect of EAW on the PSNR of the reconstructed image. Solid red line shows the results when EAW was included and dashed blue line shows the reconstructed image results when EAW is not included.

To examine the effects of EAW on measurement noise, 128 equiangular projections through the Shepp-Logan phantom were computed and Poisson noise was added to the projections.  Figure 9 shows the effect of EAW inclusion for different input peak signal-to-noise ratios (PSNR) on the PSNR of the reconstructed images. Images were reconstructed with the proposed method once with including EAW that is calculated by (9) and once without EAW. As can be seen, the PSNR is improved when the input noise is larger (small input PSNR) and its effect is less when the noise is low (larger input PSNR). The input PSNR was measured from the FBP reconstructed images.

**Figure 10:**
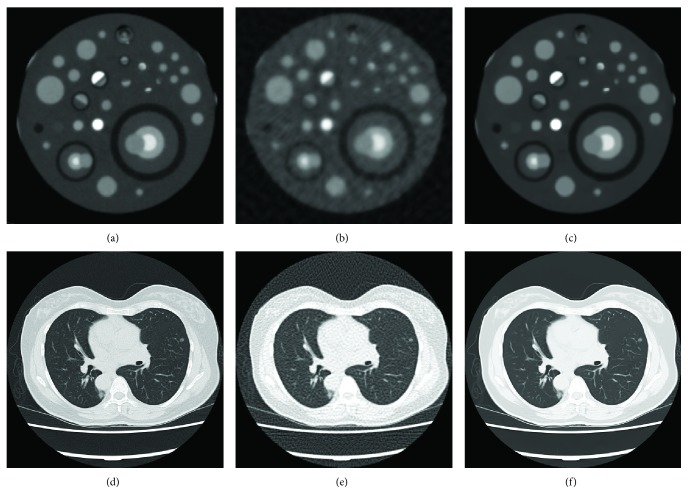
Comparison of FBP and the proposed method for the custom built cardiac plaque phantom (a–c) and from a patient chest CT scan (d–f). (a, d) Image reconstructed from 900 projections with FBP, (b, e) image reconstructed from 200 projections with FBP, and (c, f) image reconstructed from 200 projections with the proposed method.

Results obtained from the custom fabricated cardiac plaque phantom are shown in Figures 10(a)–10(c), which provides a comparison of reconstructions using FBP from 900 projections with the results obtained from 200 equiangular projections. It should be noted that the number of projection is chosen based on Figure 8, which shows that the error of the images reconstructed from 200 projections is in an acceptable range. Qualitative image evaluation was performed using a continuous linear scale of 1–5 (Excellent). Statistical analysis was performed using the paired Student *t*-test, which showed no statistical difference in image quality between the cardiac phantom images reconstructed from 200 projections using the proposed method and the full projection FBP images (*p* = 0.05).

Finally, Figures 10(d)–10(f) show reconstructions of a chest CT scan from a hospital patient using FBP from 900 projections and the proposed method with 200 projections. The image reconstructed with the proposed method has almost the same quality as the FBP. Some small details in bony structures and flat lung regions are removed or have decreased contrast in the image reconstructed by the proposed method. However, all the important details are preserved. Thus, in comparison with FBP, the proposed method uses about four times less projections; that is, the radiation dose is decreased by more than a factor of four (78% dose reduction).

**Figure 11:**
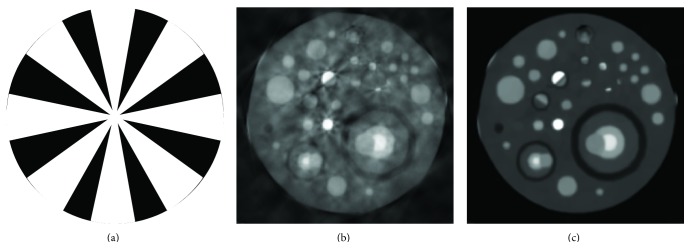
Comparison of FBP and the modified proposed method for the cardiac plaque phantom reconstructed using the protocol shown in (a), in which the projections are taken within the white areas, (b) FBP reconstructed image from the 450 projections gathered from the mask shown in image (a), and (c) image reconstructed with the modified proposed method from projections shown in image (a).

Current commercial CT scanners are unable to switch their X-ray sources on and off fast enough to achieve the proposed equiangular simulations. To overcome this problem, the mask used in Figure 11(a) is used that addresses this concern by turning the X-ray source off in the black areas over a range of angles and then turning it on in the white areas. To reconstruct high quality CT images scanned by this protocol, the reconstruction algorithm is modified by stacking the similar patches into 3D stacks [50]. Applying 3D wavelet thresholding/shrinkage on the 3D stacks of the similar patches increases the sparsity of the wavelet coefficients, which in turn improves the image reconstruction. The similar patches are selected from overlapped 15 × 15 neighborhoods and the patches are 6 × 6.

## 8. Conclusion

It has been shown that CT reconstruction can be statistically modeled as a weighted compressed sensing optimization problem. Our proposed weighted CS-based CT reconstruction algorithm was derived from the MAP model of CT imaging, considering the sparsity of the wavelet coefficients and piecewise constant nature of the CT images. Subsequently, a fast CS recovery method was proposed in which the pseudopolar based Radon transform was used as the measurement function to reduce the computational complexity. Moreover, to reconstruct CT images from nonparallel projections, rebinning to parallel beams was used. To remove the interpolation error caused by rebinning and the measurement noise, a weighting approach (EAW) was proposed. This enabled CT images to be reconstructed from a reduced number of projections. It was shown that using EAW improves the reconstruction quality substantially. For instance, a lung CT image was reconstructed with 78% lower dose but the same diagnostic quality as the image reconstructed by FBP from full data. The greatly reduced computational complexity of the proposed algorithm enabled a 512 × 512 image to be reconstructed in less than 30 sec on a desktop computer without numerical optimizations. Thus, our proposed method may be among the first CS-based CT reconstruction methods whose computational complexity is sufficiently small to enable low-dose image reconstructions to be performed without using either time-consuming computations or a complex computational system. Finally, it should be noted that the proposed method can be extended to 3D geometries by using the approaches described in Appendix A.

## Figures and Tables

**Figure 12:**
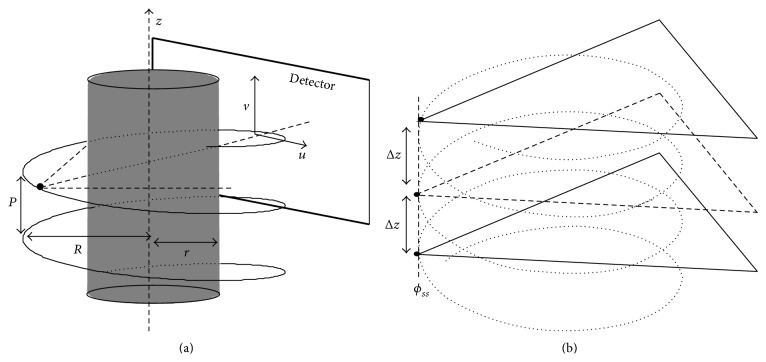
(a) Helical trajectory (adapted from Figure  1 in [52]) and (b) the fan beams in parallel *z*-slices.

**Figure 13:**
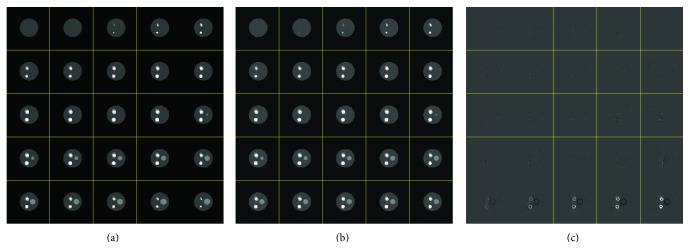
Helical scan tested on a simple simulated phantom. Pitch factor is 0.5 in this phantom data. (a) The original phantom. (b) Image reconstructed with the proposed method. (c) Difference between the true image and the reconstructed image.

**Figure 14:**
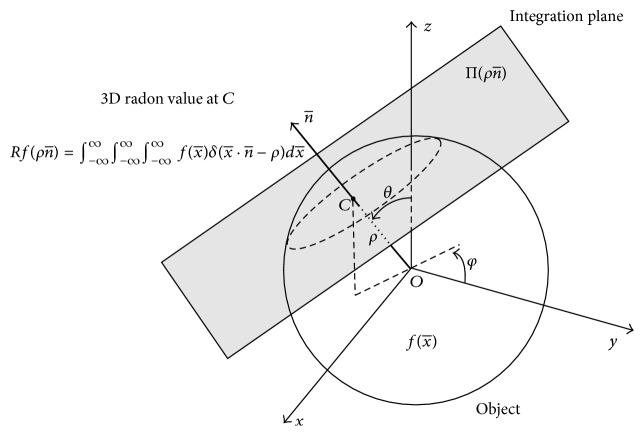
3D Radon transform of an object $f(\bar{x})$ at each point *C* is the integral of a plain passing through *C* and orthogonal to the vector that connects the point to the origin, reproduced, with permission from [51].

**Figure 15:**
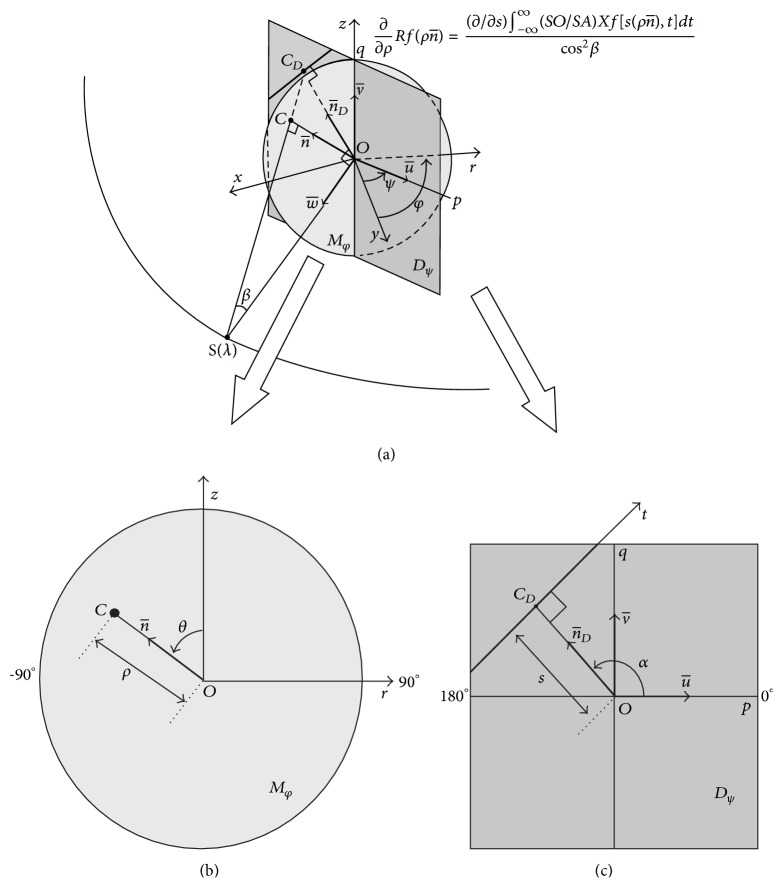
The Grangeat equation and the parameters used in it, reproduced, with permission from [51].

**Figure 16:**
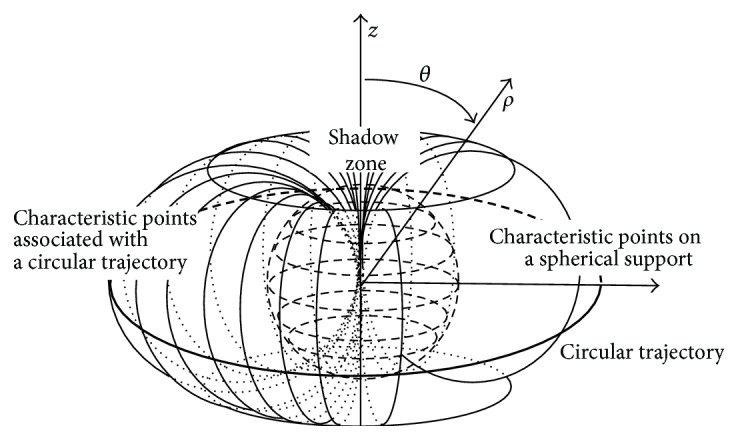
Shadow zone in the radon space associated with a circular scanning trajectory, reproduced, with permission from [51].

**Algorithm 1:**
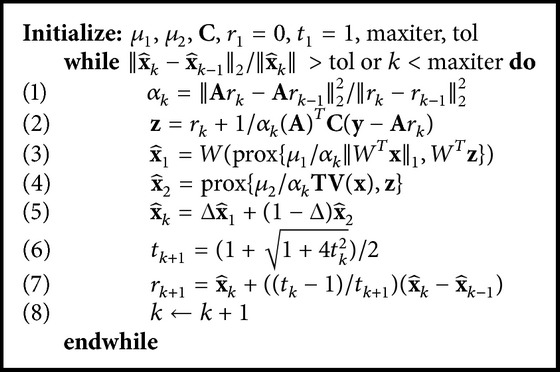
Algorithm used to solve (19).

**Algorithm 2:**
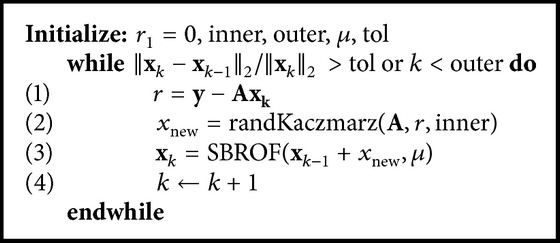
Pseudo-code of ART-TV based method used in simulations.

## Appendix

### A. Extending the Algorithm to 3-Dimensional Geometries

As discussed in Section 2.1.1, to be able to use our proposed method for nonparallel geometries, the projections should be transformed to the Radon transform data. Simple rebinnging algorithms can be used for fan beam and helical cone beam projections to redistribute the diverging beams into parallel beams, for which the Radon transform and the 2D projections are identical. However, this transformation is more complicated in 3D cases, since the 3D Radon is very different from the 3D X-ray projections.

In this appendix, we first describe a method, using which the helical cone-beam projections can be transformed to fan bean projections. This rebinning algorithm can be used in our proposed method to reconstruct 3D images from the helical cone beam projections.

In the second part, we describe a method that converts the 3D cone beam projections to 3D Radon transform. The 3D Radon transform of the object can be used in our proposed method along with 3D PPFT transform to reconstruct the cone beam images. This step is based on Grangeat's [51] formula, which can be used in our proposed method to reconstruct the 3D cone beam images.

#### A.1. Helical Cone Beam Reconstruction and Preliminary Results

To reconstruct the helically scanned objects, the scanned cone beam data should first be converted to fan-beam data and then to parallel beams. This rebinning process is based on the method introduced in [52], called cone beam single slice rebinning (CB-SSRB). CB-SSRB consists of the following two steps:

(1) For each source position in the helical trajectory, *ψ*, fix the *z*-sampling distances.

(2) For each *z*-slice, calculate the complete fan-beam set, from which the image can be estimated. This step uses the following equation to interpolate the cone beam scanned data on the fan beam points of interest:

(A.1) pzφ,u≃u2+D2u2+v2+D2gψu,v,v=u2+D2RDΔz,

where *p*_*z*_(*φ*, *u*) is estimated fan beam projection at source angle *φ* and axial position *z*, *g*_*ψ*_(*u*, *v*) is the cone beam projections at helical position, *D* is the distance between the source and the origin of the detector, and *u*, *v*, and *R* are geometry parameters defined in Figure 12. Each interpolated fan beam projection is weighted by

(A.2) wϕss,u=sin2⁡πϕss22γT+2γif  ϕss∈0,2γT+2γ1if  ϕss∈2γT+2γ,π+2γsin2⁡ππ+2γT−ϕss22γT−2γif  ϕss∈π+2γ,π+2γT,

where *ϕ*_*ss*_ = (*π*/2 + *γ*_*T*_)(1 − Δ*z*/*d*), *d* = 0.5*P*(*π*/2 + *γ*_*T*_)/(2*π*), *P* is the pitch of the helical trajectory, and 2*γ*_*T*_ is the maximum fan angle. The parallel beams *g*(*l*, *φ*) can then be estimated from the weighted fan beams *p*_*z*_(*φ*, *u*) using (4), from which **y** will be calculated by computing the 1D Fourier transform of *g*(*l*, *φ*)s.  Figure 13 shows a simple simulated phantom reconstructed by the proposed helical reconstruction method. The helix source position is defined as *ψ* = [*R*cos(*φ*), *R*sin(*φ*), *P*(*φ*/2*π*)] and in this test pitch factor *P* = 0.5. As can be seen, aside from the start or end of the scan, the reconstruction is almost perfect. However, when the image is close to one of the endpoints, the error of rebinning increases and as a result the image reconstruction error increases.

#### A.2. Future Work: Volumetric Cone-Beam CT

While in 2D geometries the X-ray projection and the Radon transform can be used alternately, in higher dimensions (e.g., cone beam geometry) the Radon transform and X-ray projections are very different. This makes it impossible to use inverse Radon transform and Fourier slice theorem to reconstruct 3D images from the X-ray projections, directly. The 3D Radon transform of a 3D function *f*(·), shown in Figure 14, is defined as

(A.3) $$\begin{array}{c} Rf\left(\;\rho \bar{n}\;\right)=\overset{\infty }{\underset{-\infty }{\int }}\overset{\infty }{\underset{-\infty }{\int }}\overset{\infty }{\underset{-\infty }{\int }}f\left(\bar{x}\right)\delta \left(\bar{x}\cdot \bar{n}-\rho \right)d\bar{x}, \end{array}$$

where $\bar{n}$ is the unit vector that passes through the origin and the point of interest, which is described by (*ρ*, *θ*, *φ*) in spherical coordinate and $\bar{x}=(x,y,z)$. This means that the Radon transform of an object *f* at a point is equal to the integral of the object on a plane that passes the point and is normal to the vector that connects the origin to that point.

The Grangeat formula relates line integral of cone-beam data to the Radon data in the whole Radon space for exact image reconstruction [51]. The link between the 3D radon *Rf* and the X-ray projections can be expressed as follows:

(A.4) $$\begin{array}{c} \frac{\partial }{\partial \rho }Rf\left(\;\rho \bar{n}\;\right)=\frac{1}{\cos^{2}\beta }\frac{\partial }{\partial s}\overset{\infty }{\underset{-\infty }{\int }}\frac{SO}{SA}Xf\left[\;s\left(\;\rho \bar{n}\;\right),t\;\right]dt, \end{array}$$

where $Xf[s(\rho \bar{n}),t]$ is the detector value at a distance of *s* from the center of detector *O* along the line *t* perpendicular to *OC*_*D*_ (shown in Figure 15), *SO* denotes the source to origin distance, and *SA* is the distance between the source and an arbitrary point *A* along line *t*. Other parameters are defined in Figure 15.

To have an exact reconstruction, the Grangeat formula requires the sources on a curve to contain at least one source at each plane meeting support of the object, *f*(·). This condition is obviously not satisfied for a plane circle for which the FDK approximation has been derived. As shown in Figure 16, some data needed for exact image reconstruction are missed in this geometry. The missed data can be estimated using compressed sensing and the error adaptation weights proposed in this paper.

To reconstruct the cone beam images, the following process is proposed. Using (A.4), the radial derivatives of the 3D Radon transform of the object are estimated on 3D pseudopolar grids. The 3D pseudopolar grids consist of three groups of grids *P* ≡ *P*_1_ ∪ *P*_2_ ∪ *P*_3_:

(A.5) P1=m,−2kNm,−2lNm,P2=−2kNm,m,−2lNm,P3=−2kNm,−2lNm,m,

where *k*, *l* = *N*/2,…, *N*/2, *m* = 3*N*/2,…, 3*N*/2, and *N* is the number of pixels of the 3D function in each dimension. Similar to the 2D case, a fast 3D pseudopolar Fourier transform is available [53] enabling a 3D object *f*(·) to be reconstructed from the Fourier transform of the derivatives of the estimated 3D Radon transform. The interpolation errors caused by numerical implementation of the Grangeat equation are tracked and included in the EAW to minimize their effect in the recovered images and to enable the optimization algorithm to recover the missed data more effectively.
